# Arterial spin labeled perfusion MRI for the assessment of radiation-treated meningiomas

**DOI:** 10.3389/fradi.2024.1345465

**Published:** 2024-03-18

**Authors:** Paul Manning, Shanmukha Srinivas, Divya S. Bolar, Matthew K. Rajaratnam, David E. Piccioni, Carrie R. McDonald, Jona A. Hattangadi-Gluth, Nikdokht Farid

**Affiliations:** ^1^ Department of Radiology, University of California, San Diego, San Diego, CA, United States; ^2^ Center for Multimodal Imaging and Genetics, University of California, San Diego, San Diego, CA, United States; ^3^ Center for Functional Magnetic Resonance Imaging, University of California, San Diego, San Diego, CA, United States; ^4^ Department of Neurosciences, University of California, San Diego, San Diego, CA, United States; ^5^ Department of Psychiatry, University of California, San Diego, San Diego, CA, United States; ^6^ Department of Radiation Medicine and Applied Sciences, University of California, San Diego, San Diego, CA, United States

**Keywords:** ASL, arterial spin labeled, perfusion, CBF, cerebral blood flow, meningioma

## Abstract

**Purpose:**

Conventional contrast-enhanced MRI is currently the primary imaging technique used to evaluate radiation treatment response in meningiomas. However, newer perfusion-weighted MRI techniques, such as 3D pseudocontinuous arterial spin labeling (3D pCASL) MRI, capture physiologic information beyond the structural information provided by conventional MRI and may provide additional complementary treatment response information. The purpose of this study is to assess 3D pCASL for the evaluation of radiation-treated meningiomas.

**Methods:**

Twenty patients with meningioma treated with surgical resection followed by radiation, or by radiation alone, were included in this retrospective single-institution study. Patients were evaluated with 3D pCASL and conventional contrast-enhanced MRI before and after radiation (median follow up 6.5 months). Maximum pre- and post-radiation ASL normalized cerebral blood flow (ASL-nCBF) was measured within each meningioma and radiation-treated meningioma (or residual resected and radiated meningioma), and the contrast-enhancing area was measured for each meningioma. Wilcoxon signed-rank tests were used to compare pre- and post-radiation ASL-nCBF and pre- and post-radiation area.

**Results:**

All treated meningiomas demonstrated decreased ASL-nCBF following radiation (*p* < 0.001). Meningioma contrast-enhancing area also decreased after radiation (*p* = 0.008) but only for approximately half of the meningiomas (9), while half (10) remained stable. A larger effect size (Wilcoxon signed-rank effect size) was seen for ASL-nCBF measurements (*r* = 0.877) compared to contrast-enhanced area measurements (*r* = 0.597).

**Conclusions:**

ASL perfusion may provide complementary treatment response information in radiation-treated meningiomas. This complementary information could aid clinical decision-making and provide an additional endpoint for clinical trials.

## Introduction

Meningiomas are the most common primary brain tumors in the United States, accounting for 36.1% of all primary brain tumors (1). A variety of treatment options exist for management of meningiomas including surgical resection, radiotherapy, or a combination of the two (2–6). Of these treatment options, radiotherapy can be particularly beneficial for meningiomas in difficult to access sites such as the skull base or for meningiomas intimately associated with critical structures. Numerous studies have shown high rates of long-term local control using radiotherapy as the primary treatment modality or as an adjunct to surgery (3, 4, 7–10).

An ongoing challenge with radiation treatment for meningiomas is evaluation of post-radiation treatment response (6, 11). Prior studies evaluating meningioma radiation treatment response have relied on variations of the Macdonald criteria (12–14) (designed for high-grade glioma treatment response) or the Response Evaluation Criteria in Solid Tumors (RECIST) (15) (designed for systemic tumor treatment response), but neither of these criteria fully account for effects specific to meningiomas, nor radiation response. Recently, the Response Assessment in Neuro-oncology (RANO) Working Group released a report proposing standardized response assessment criteria for meningiomas (16). These guidelines allow for improved standardization of meningioma treatment response assessment, but there are persistent inherent difficulties. Conventional contrast-enhanced MRI size measurements remain the primary radiologic tool for assessment of meningioma response or progression; however, even when meningiomas are effectively treated with radiation, they often demonstrate little to no change in size (17–19). Additionally, slowly progressive meningiomas may not demonstrate a meaningful size increase for several years after failed treatment (11, 16). Given these limitations, novel endpoints for meningioma treatment response are being sought which would provide more immediate and quantitative evaluation, thereby aiding clinical decision making and allowing for improved standardization of clinical trial endpoints.

Arterial spin labeling (ASL) is a promising MR perfusion-weighted imaging technique which may be useful for evaluation of radiation treatment response in meningiomas. In ASL imaging, inflowing arterial blood is magnetically “labeled” through the use of radiofrequency inversion pulses, allowing for quantitative tissue perfusion measurements (20–22). Because ASL evaluates tissue perfusion, ASL may provide a way to evaluate physiologic treatment response beyond the structural information provided by conventional contrast-enhanced MRI. Previous studies have shown that in meningiomas, quantitative ASL perfusion measurements correlate with histologic measures of micro-vessel area (23) and micro-vascular density (24). Based on these prior studies and known mechanisms of radiation treatment effects including endothelial cell damage, small vessel injury, decreased capillary perfusion, and decreased micro-vascular density (25–28), we hypothesize that ASL perfusion may provide an effective technique to capture the microvascular changes that occur in meningiomas after radiation treatment. No other studies have assessed ASL for meningioma radiation treatment response. Compared to other proposed techniques, such as PET-based techniques (29, 30), ASL does not require a separate PET tracer, additional scan, or additional contrast agent. Additionally, ASL can be integrated into routine follow up MR exams, thus providing an attractive technique to efficiently monitor meningioma treatment response.

Therefore, the purpose of this study is to assess ASL perfusion for the evaluation of radiation treatment response in meningiomas.

## Materials and methods

### Study design and patients

This retrospective, observational, single-institution study was approved by an Institutional Review Board and was compliant with the Health Insurance Portability and Accountability Act. Between June 2014 and March 2021, thirty-two adults at our institution were identified who received radiation treatment for meningiomas and received clinical MR evaluation before and after radiation. From this group, twenty patients were selected who met the following inclusion criteria: (i) confirmed diagnosis of meningioma by biopsy or resection, or by consensus imaging and clinical criteria; (ii) MRI of the brain including post-contrast and 3D pseudocontinuous arterial spin labeled (3D pCASL) sequences before and after radiation (9 patients were excluded because 3D pCASL was not performed either before or after radiation); (iii) residual measurable disease following resection, measuring at least 5 mm in two perpendicular dimensions (2 patients were excluded because no measurable disease was present after resection); (iv) perfusion was measurable on the baseline pre-radiation MRI (1 patient was excluded because perfusion was not measurable in a thin en-plaque meningioma); (v) standard of care radiotherapy including either stereotactic radiosurgery (SRS), stereotactic radiation therapy (SRT), or external beam radiation therapy (EBRT).

### MR exams

All MR exams were performed on a 3 T MRI scanner (Discovery 750, GE Healthcare, Milwaukee Wisconsin) using an 8-channel brain array coil. Conventional MRI protocol included post-contrast 3D T1-weighted fast spoiled gradient-echo (FSPGR) imaging (TE/TR = 3.0/6.9 ms; FA = 9°; FOV = 25 cm; matrix = 256 × 256; slices = 180, slice thickness = 1 mm, interslice gap 1 mm). Contrast enhanced exams were performed using either gadobenate dimeglumine (Bracco Diagnostics) or gadobutrol (Bayer AG), both at 0.1 mmol/kg.

ASL was performed using pseudocontinuous labeling with a 3D stack-of-spirals fast spin echo readout; this reflects the GE product ASL sequence. PCASL-specific parameters included a labeling duration of 1,450 ms and post labeling delay of 2025 ms with 3D spiral readout parameters as follows: spiral interleaves = 8; points per spiral = 512; slices = 36; slice thickness 4.0–4.2 mm; FOV = 24–26 cm; in-plane resolution = 3.64–4.53 mm^2^; bandwidth = 62.5 kHz; TE = 9.5–10.5 ms; TR = 4,800–4,847 ms; NEX = 3; and scan time = 4 min 32 s–4 min 42 s.

### Image analysis

Maps of ASL-derived cerebral blood flow (ASL-CBF) were generated from the 3D pCASL images using ReadyView ASL (GE Healthcare). ASL-CBF maps were co-localized with post-contrast T1-weighted images. A board-certified neuroradiologist with 10 years of experience (NF) and a neuroradiology fellow with 5 years of experience (PM), who were blinded to patient clinical and pathologic data, reviewed each ASL perfusion map and deemed to be sufficient diagnostic quality to assess perfusion within the meningioma. Subsequently, both readers placed circular ROIs on the regions of maximum perfusion signal on the CBF maps, within a slice, corresponding to the area of meningioma contrast enhancement on the co-localized post-contrast T1-weighted images. Areas of necrosis, surgical cavities, vessels, hemorrhage, and susceptibility artifact were avoided. To normalize the CBF values, the cerebellum was chosen as the reference region. The cerebellum was chosen because meningiomas are extra-axial tumors; therefore, normalization to traditional contralateral “normal appearing” gray or white matter (used for intra-axial tumors) is less applicable. Several prior studies have described the cerebellum as a useful reference region for the evaluation of normalized CBF including studies of gliomas (31) and meningiomas (32). Accordingly, to evaluate normalized CBF perfusion values (ASL-nCBF), an additional ROI was placed in the ipsilateral cerebellar hemisphere measuring approximately 2 × 2 cm capturing an average distribution of gray and white matter, and normalized perfusion values were calculated by dividing the signal intensity within the meningioma ROI by the signal intensity within the cerebellar ROI.

Per the RANO meningioma response assessment guidelines (16), meningioma size estimates were made by manually measuring the maximum meningioma diameter in two perpendicular planes on the axial post-contrast 3D FSPGR images. Estimates of tumor area were calculated by multiplying the perpendicular diameter measurements. Measurements were made by an image analyst with 2 years of experience (SS) and were approved by the board-certified neuroradiologist (NF).

### Statistical analysis

Statistical analyses were performed using R version 3.6.1 (R Core Team, 2019). For patients evaluated over multiple time points, the pre-radiation and the most recent post-radiation ASL perfusion time points were evaluated. Wilcoxon signed-rank tests were used to evaluate differences in ASL perfusion before and after radiotherapy as well as differences in contrast enhancing area before and after radiotherapy. A *p*-value of 0.05 was considered statistically significant. For patients with histologic data (15 out of 20 patients), group differences between low-grade (grade I) and higher-grade (grade II or III) meningiomas were evaluated for pre-radiation ASL perfusion. Group differences were tested using Mann-Whitney U tests. Finally, inter-reader agreement was evaluated by calculating the intraclass correlation coefficient (ICC) for the two readers.

## Results

### Patient population

Twenty adults with radiation-treated meningiomas (12 female and 8 male) met inclusion criteria, and ages ranged from 27 to 72 years (mean 54 ± 12 years). Histologic data was available for 15 out of 20 patients. Of patients with histologic data, 7 patients were diagnosed with WHO grade I, 5 patients with WHO grade II, and 3 patients with WHO grade III meningioma. All patients were treated with radiotherapy: 4 patients were treated with SRS (dose ranging from 2,100–2,400 cGy in 3 fractions) and 16 were treated with EBRT (dose ranging from 5,400–6,600 cGy in 30–33 fractions). Most patients underwent either subtotal resection or near gross total resection prior to radiotherapy (15 patients), but 5 patients were treated with radiotherapy alone. The post-radiation evaluations were performed between 2 and 35 months (median 6.5 months) after patients completed radiotherapy. Descriptive information regarding the study cohort is provided in Table 1.

**Table 1:** Descriptive characteristics of patient cohort.

Patient number	Age	Sex	Grade	Resection	Radiotherapy	Radiotherapy total dose (cGy)	Time between radiotherapy and post ASL exam (months)
1	61	M	2	nGTR	EBRT	5,400	6
2	65	M	3	STR	EBRT	6,600	2
3	47	F	1	nGTR	EBRT	5,400	6
4	60	F	3	STR	EBRT	6,600	5
5	27	M	NA	None	EBRT	5,580	6
6	67	F	1	STR	EBRT	5,400	6
7	45	F	2	STR	EBRT	5,850	7
8	50	F	3	STR	EBRT	6,000	7
9	29	F	2	nGTR	EBRT	6,000	6
10	42	F	1	STR	EBRT	5,400	12
11	58	F	1	STR	SRS	2,400	3
12	67	M	NA	None	SRS	2,400	11
13	72	F	2	STR	EBRT	5,940	6
14	60	F	NA	None	SRS	2,400	13
15	55	M	1	STR	EBRT	5,940	6
16	54	M	NA	None	SRS	2,100	18
17	56	F	1	STR	EBRT	5,400	20
18	46	F	1	STR	EBRT	5,580	25
19	52	M	2	STR	EBRT	5,625	22
20	67	M	NA	None	EBRT	5,400	35

M, male; F, female; nGTR, near gross total resection; STR, subtotal resection; EBRT, external beam radiotherapy; SRS, stereotactic radiosurgery; cGy, centigray.

Grade = meningioma WHO Grade I, II, or III.

### Quantitative ASL perfusion and contrast-enhancing area analysis

**Figure 1:**
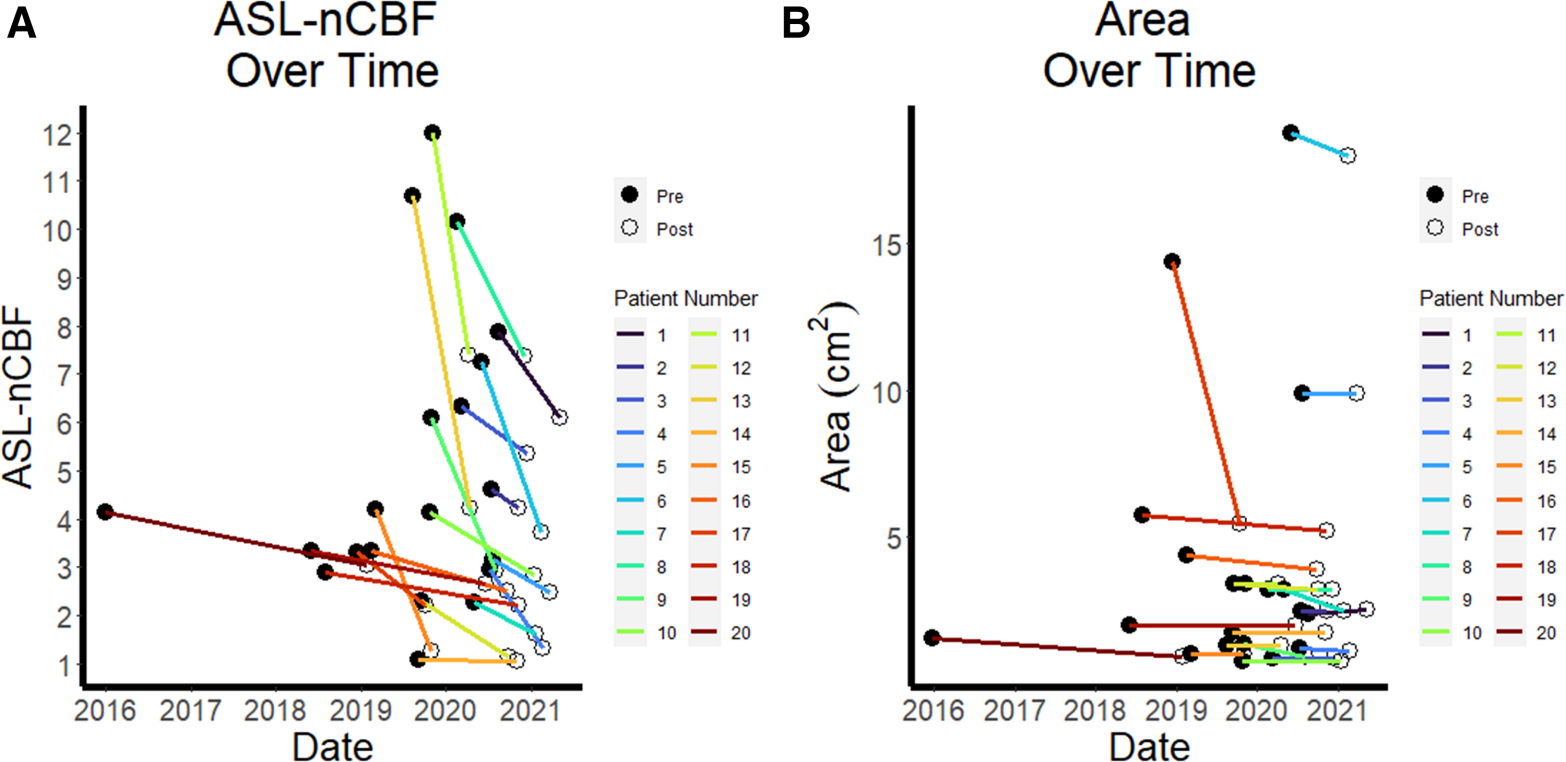
ASL perfusion and size over time. Line plots demonstrate the pre- and post-radiation normalized ASL cerebral blood flow (ASL-nCBF) values for each patient over time, with a general downward trend (**A**) Comparison line plots demonstrate the pre- and post-radiation contrast-enhanced area measurements for each patient over time, also with a slight downward trend, but less pronounced than for ASL-nCBF (**B**).

**Figure 2:**
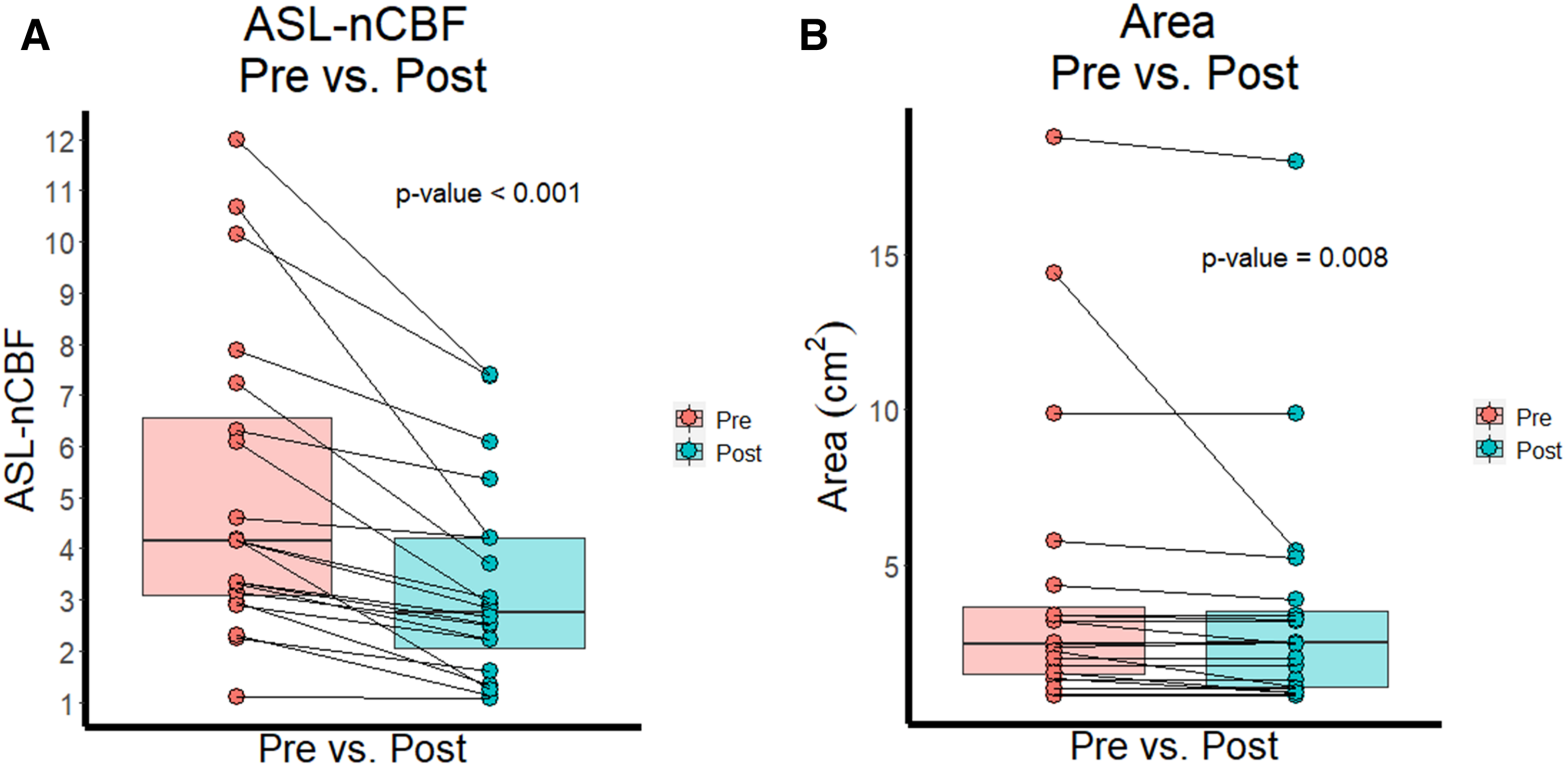
ASL perfusion and size before and after radiation. Box plots and paired line plots display differences between pre- and post-radiation ASL-nCBF (**A**) Comparison box plots and paired line plots display differences between pre- and post-radiation contrast-enhanced area (**B**) Differences were significant for both ASL-nCBF and area measurements, but the effect size was larger for ASL-nCBF.

Both ASL-nCBF and contrast enhancing area measurements decreased after radiation (Figure 1). Post-radiation ASL-nCBF was significantly lower than pre-radiation ASL-nCBF (*p* < 0.001) with median pre-radiation ASL-nCBF measuring 4.1 (IQR: 3.1–6.6) and median post-radiation ASL-nCBF measuring 2.7 (IQR: 2.1–4.2) (Figure 2). The effect size (Wilcoxon signed-rank effect size) between pre- and post-radiation ASL-nCBF measurements was large (*r* = 0.877). Similarly, post-radiation contrast-enhanced size measurements were also lower than pre-radiation contrast-enhanced size measurements (*p* = 0.008), with median pre-radiation area measuring 2.4 (IQR: 1.5–3.6) cm^2^ and median post-radiation area measuring 2.5 (IQR: 1.1–3.5) cm^2^ (Figure 2). Although the median pre- and post-radiation areas were nearly the same, the average and interquartile ranges were lower following radiation. The effect size between pre- and post-radiation contrast-enhanced size measurements was not as large (*r* = 0.597) compared to the effect size between pre- and post-radiation ASL-nCBF measurements (*r* = 0.877).

**Figure 3:**
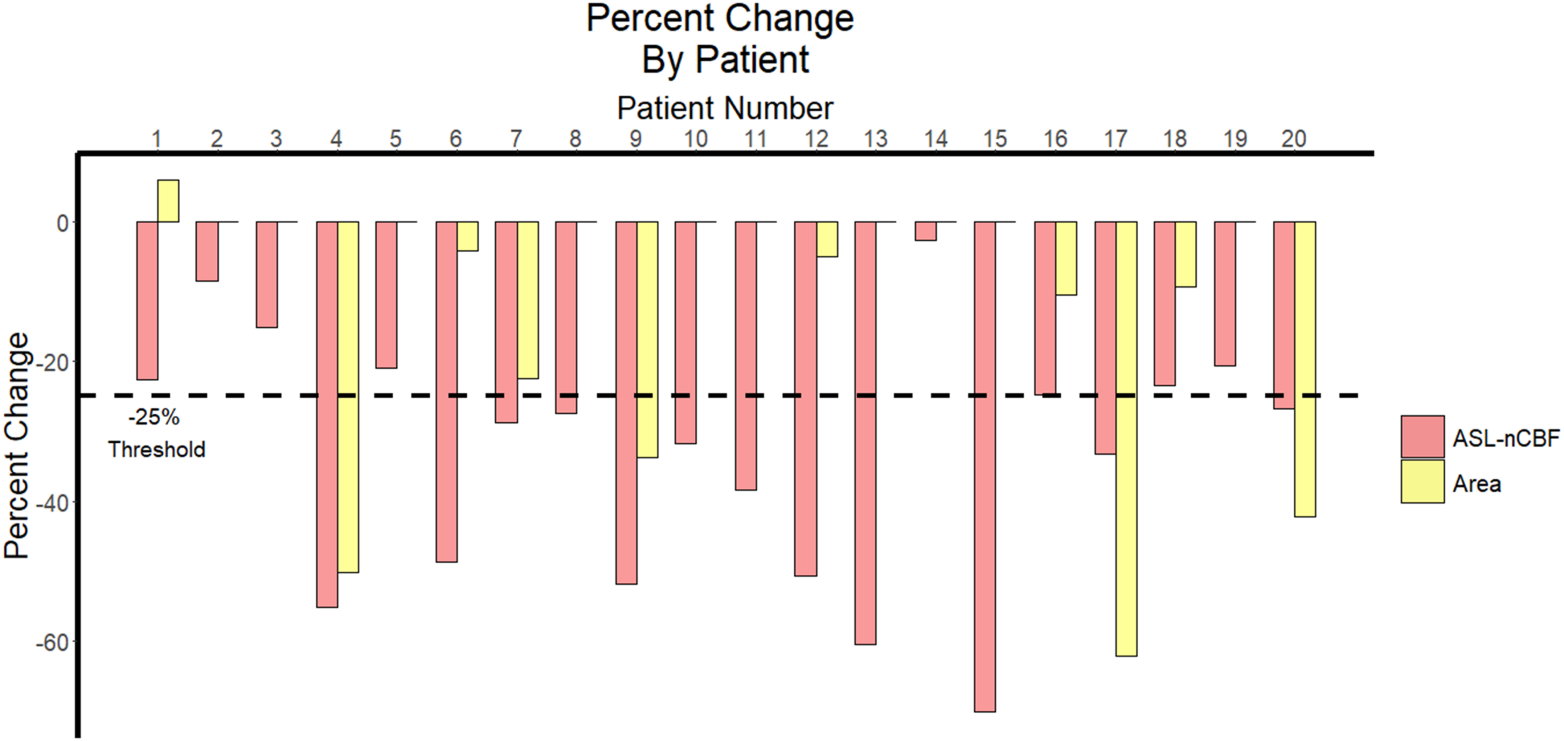
Percent change in ASL perfusion and size following radiation. Bar plot demonstrates the percent change between pre- and post-radiation ASL-nCBF and contrast-enhanced area measurements for each patient. The 25% threshold used by RANO criteria to indicate meaningful change is highlighted with a dashed horizontal line.

All patients demonstrated decreased ASL-nCBF after radiation. However, for contrast-enhanced area measurements, 9 patients demonstrated a decrease in size, 10 patients demonstrated no change, and 1 patient demonstrated a small increase in size. Furthermore, twelve patients demonstrated a decrease in ASL-nCBF measuring at least 25%, whereas only 4 patients demonstrated a decrease in size measuring at least 25% (Figure 3).

No significant change was seen between the low-grade and higher-grade histology groups for pre-radiation ASL-nCBF measurements (*p* = 0.698).

The inter-reader agreement was excellent with ICC measuring 0.975.

### Illustrative case

**Figure 4:**
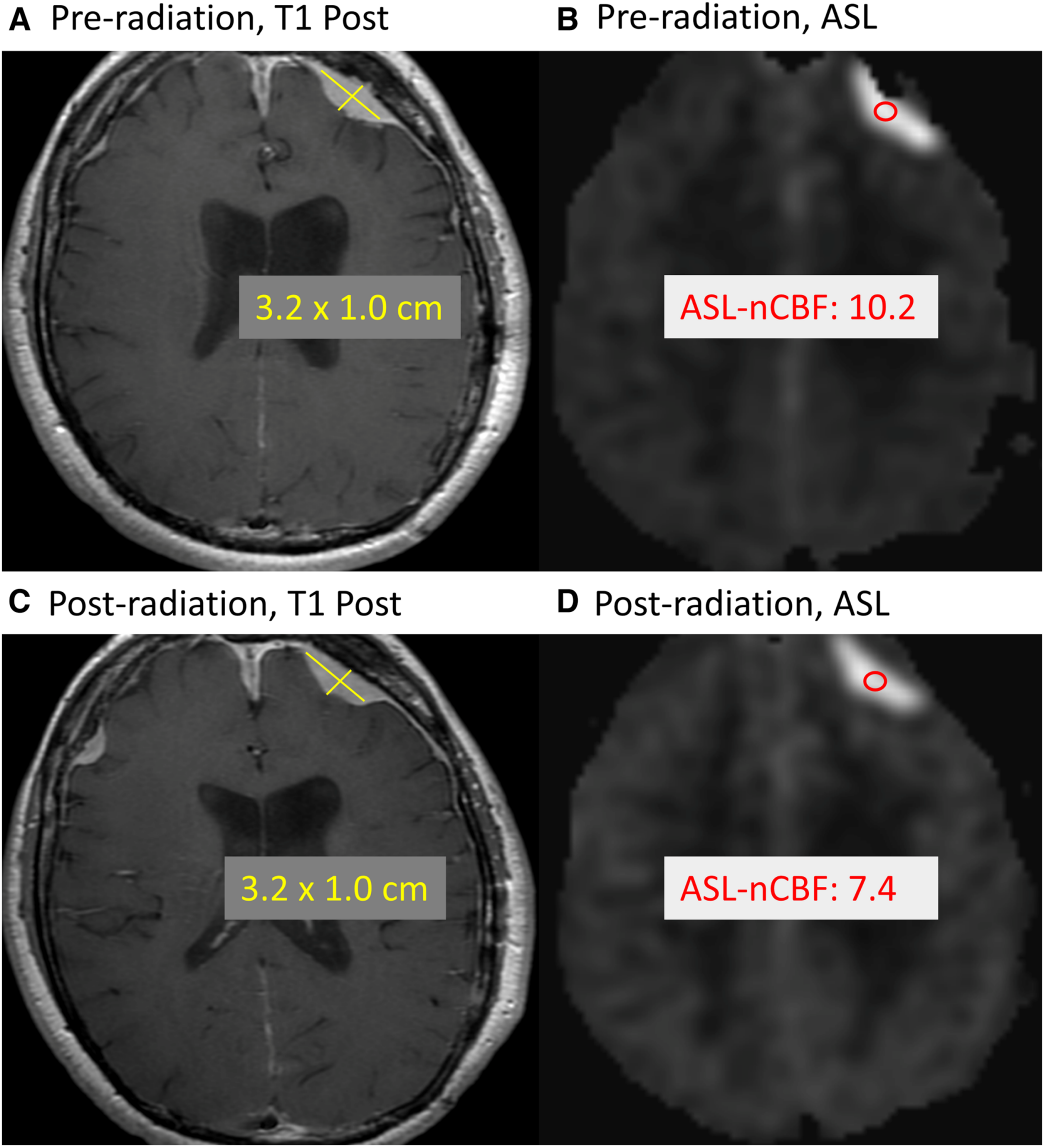
Example of treated meningioma. Example case demonstrates a meningioma along the left frontal convexity before and after radiation treatment. Comparing the pre-radiation contrast-enhanced size (**A**) and ASL-nCBF measurements (**B**) to post-radiation size (**C**) and ASL-nCBF measurements (**D**), the size remains stable while ASL-nCBF decreases.

In Figure 4, we highlight a representative case which demonstrates a decrease in ASL-nCBF on the post-radiation scan, with no appreciable change in meningioma size.

## Discussion

To our knowledge, no other studies have assessed ASL for the evaluation of radiation treatment response. In this initial study, we found that all meningiomas demonstrated decreased ASL-nCBF after radiation, with a relatively large effect size. Contrast-enhanced area also decreased after radiation, but only in approximately half of the meningiomas and with a smaller effect size. These findings suggest that ASL perfusion measurements may provide an additional complementary quantitative method for evaluating post-radiation treatment response in meningiomas, particularly in cases where conventional contrast-enhanced area measurements show no change. We suspect the reason we observed a larger effect size for ASL perfusion measurements compared to conventional contrast-enhanced area measurements is because ASL captures physiologic response to radiation treatment, whereas contrast-enhanced area measurements predominantly capture structural information.

Currently, the efficacy of radiation treatment in meningiomas is determined by stability or decrease in size over time. However, slow-growing and indolent meningiomas may not demonstrate a meaningful size increase for several years after failed treatment. Therefore, using size measurements alone, effectively treated meningiomas may not be accurately discriminated from progressive meningiomas for months or years after treatment. ASL may provide early quantitative evaluation of radiation treatment response which may inform clinical decisions sooner, including decisions regarding how long to wait before repeat imaging, whether to continue observation, or whether to re-resect or re-irradiate. Additionally, ASL may provide complementary information for clinical trial endpoints. Because meningiomas are very slow growing neoplasms, most meningioma clinical trials rely on surrogate endpoints such as progression-free survival at 6 months (PFS6) or radiographic objective response rate (ORR) rather than overall survival (OS) (16). However, these surrogate endpoints depend on contrast-enhanced size measurements which may be stable over the first 6 months following treatment, despite slow progression, or may not show enough change to be considered significant by radiologic ORR criteria (16). Early quantitative evaluation of radiation treatment response using ASL could provide complementary treatment information in clinical trials when size measurements remain stable.

Our results parallel previous short-term and subsequent long-term studies of the metabolic PET agent ^11^C-L-methionine for evaluation of radiation treatment response in meningiomas (29, 30). Similar to our study, the short-term study of ^11^C-L-methionine demonstrated that early after radiation (within the first 36 months after treatment), most meningiomas demonstrated decreased uptake of ^11^C-L-methionine while contrast-enhanced size measurements showed little change, suggesting that ^11^C-L-methionine PET imaging may provide a way to detect early post-radiation treatment response. The subsequent long-term study of ^11^C-L-methionine (10 year follow up after treatment) showed that although most meningiomas demonstrated decreased uptake early after treatment, this initial decrease was only modestly predictive of later progression. A similar long-term study of ASL perfusion would be necessary to determine whether early post-radiation ASL-nCBF changes are predictive of long-term response or progression. One clear benefit of ASL compared to ^11^C-L-methionine is that the 3D pCASL sequence can be added directly to routine follow up MR exams without separate procedures for radiotracer synthesis, injection, and image acquisition which are necessary for ^11^C-L-methionine PET imaging. Thus, ASL imaging could be more ubiquitously applied for monitoring, would be less expensive, and would not require special contrast agents. Additionally, 3D pCASL has been shown to be reliable and repeatable over multiple brain segments and between different scanners and vendors (33, 34) suggesting a relatively robust imaging biomarker.

**Figure 5:**
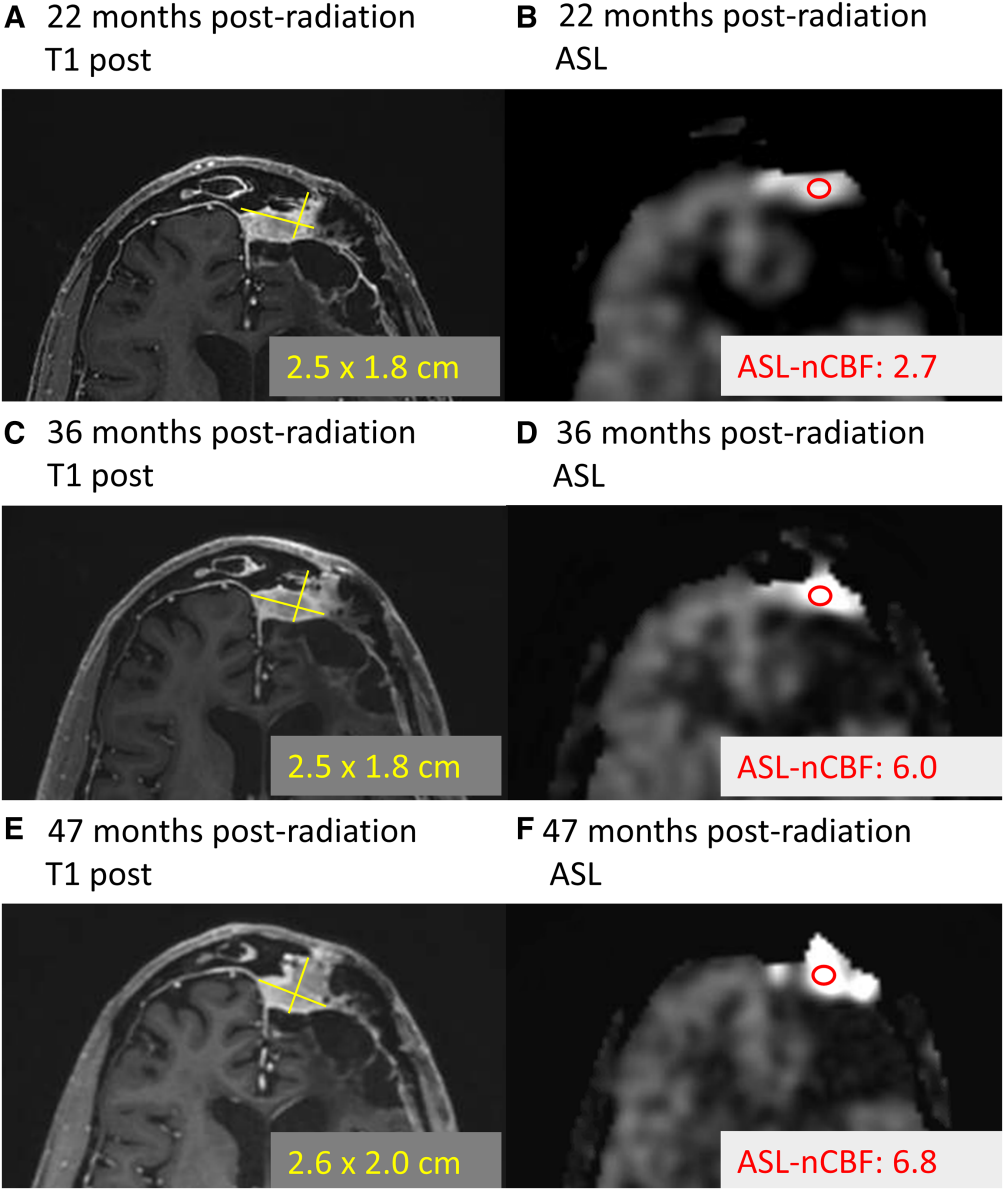
Example of progressing meningioma. Example case demonstrates a meningioma located along the left frontal convexity slowly progressing over time. At 22 months following radiation, the contrast-enhanced size (**A**) and ASL-nCBF measurements (**B**) are provided. Later, 36 months following radiation, the size remains stable (**C**), but the ASL-nCBF dramatically increases (**D**) Finally, after 47 months following radiation, the size eventually also increases (**E**), along with the ASL-nCBF (**F**).

Almost none of the meningiomas in our cohort progressed during the study window (median time to follow up 6.5 months), but this is not unexpected given that meningiomas are known to progress both rarely and slowly, with median time to progression on the order of 4.1 years for grade I meningiomas (35) and 2.1 years for grade II and III meningiomas (36). Anecdotally, one meningioma in our cohort progressed after the study window (patient 19, grade II meningioma). Review of this meningioma demonstrated that ASL-nCBF initially decreased after radiation and remained decreased at 22 months following radiation (during the study window). However, at 38 months following radiation, the ASL-nCBF subsequently increased, preceding an increase in size (Figure 5). Although anecdotal, this case suggests that ASL may provide an early indicator of progression, possibly even before size increases. This may be particularly helpful for progressing meningiomas because slow changes in size can be subtle and difficult to perceive by conventional MRI. Long-term follow up including more cases of progressive disease would be necessary to investigate these findings.

We hypothesized that ASL perfusion values would be helpful to discriminate low- from higher-grade meningiomas, but we found no significant difference in the pre-radiation perfusion values between grades. One potential explanation is that higher-grade meningiomas are more likely to out-grow their blood supply and result in necrosis and heterogenous regions of perfusion which could lead to an overall reduction in perfusion. In contrast to our study, a previous study investigating ASL perfusion in meningiomas demonstrated that ASL was able to differentiate low- from higher-grade using qualitative ASL perfusion pattern analysis rather than overall cerebral blood flow (37). A qualitative approach may better account for heterogenous perfusion patterns which may be seen in higher-grade meningiomas. Additionally, as the previous study suggested, the relationship between neo-angiogenesis and meningioma grade may not be direct as it is in gliomas. There was, however, overall variability in the pre-radiation ASL perfusion measurements between meningiomas. This is in keeping with findings described by Kimura et al. who showed that that histology patterns between meningiomas can affect the degree of ASL perfusion, highest for angiomatous meningiomas and lowest for fibrous meningiomas, potentially explaining some of these differences (23).

Unexpectedly, one case in our cohort demonstrated a significant decrease in size following radiation, with only a small decrease in ASL perfusion. Detailed review of this case revealed that the initial size was large, measuring 14.4 cm^2^ in cross-sectional area. The relatively small decrease in ASL perfusion could be due to our pre-defined ROI-measuring procedure. Based on our procedure, we placed an ROI on the area with highest ASL signal intensity, but for this large meningioma this only captured a small fraction of the lesion. Qualitatively, most of the meningioma actually demonstrated substantially decreased perfusion. This case highlights the imperfect nature of the ROI-method for perfusion evaluation. Ideally, full lesion segmentation and 3D measurements could be performed, potentially using histogram analyses to comprehensively evaluate tumor perfusion. However, we believe the ROI-method we employed is reflective of current clinical practice where dedicated histogram analyses are less likely to be performed, and we believe simple ROI-based methods are more likely to be adopted in a clinical setting.

Compared to other perfusion-weighted MRI techniques like dynamic susceptibility contrast (DSC) or dynamic contrast-enhanced (DCE) imaging, ASL may be particularly well suited for evaluation of radiation-treated meningiomas for several reasons. Meningiomas are extra-axial tumors which lack a blood-brain barrier. The absence of the blood-brain barrier can exacerbate contrast “leakiness” which can occur in contrast-based techniques, resulting in erroneously high or low quantitative values (38, 39). Since ASL is a non-contrast perfusion technique, the absence of the blood-brain barrier would not be expected to affect the perfusion values for meningiomas. Additionally, issues of nephrogenic systemic fibrosis (NSF) and gadolinium deposition inherent to contrast-based techniques (40, 41) are avoided with ASL perfusion. Finally, unlike DSC and DCE, newer implementations of ASL are spin-echo (T2) based sequences, mitigating problems related to susceptibility artifact which can be pronounced on gradient-echo (T2*) based sequences. This is particularly beneficial for evaluation of meningiomas at the skull base where treatment with radiotherapy may be preferred and susceptibility artifact can be pronounced (31, 42).

Several limitations to our study should be acknowledged. First, our study was conducted with a small sample size at a single institution. Second, although inclusion and exclusion criteria aimed to evaluate a representative cohort of patients, there is inherent heterogeneity in the cohort as described in the patient demographics table with mixed meningioma grades (including grades I, II, and III), variable initial meningioma size, variable extent of resection, and variable radiotherapy treatment type. Third, an important limitation to our study was the limited time to follow up after radiation treatment. Most of the patients in our cohort were followed for less than one year after treatment. Our intent was to evaluate early post-radiation treatment response; however, due to the slow-growing nature of meningiomas, in order to adequately evaluate whether early changes in perfusion are predictive of future meningioma recurrence or progression, a long-term follow up study would be necessary. This is already underway at our institution. Related to the limited time to follow up, only one anecdotal example of meningioma progression was captured within our study. We believe this is because most meningiomas do not progress within a short, less than one year, time frame (up to four years for grade I meningiomas). A longer time to follow up would allow more examples of progressive disease to be captured so that ASL perfusion could be evaluated in these cases.

If future multi-center and longitudinal studies support the current findings, ASL perfusion could potentially be integrated into standard meningioma imaging protocols, thereby providing complementary treatment response information beyond contrast-enhanced size measurements for clinical decision making. At our institution, ASL is now included as part of the standard of care protocol for evaluation of meningiomas before and after radiation.

## Conclusion

ASL perfusion may provide an early complementary quantitative measure of treatment response in radiation-treated meningiomas. This complementary information could aid clinical decision-making and provide an additional endpoint for clinical trials.

## Data Availability

The raw data supporting the conclusions of this article will be made available by the authors, without undue reservation.
